# Flying Real-Time Network to Coordinate Disaster Relief Activities in Urban Areas †

**DOI:** 10.3390/s18051662

**Published:** 2018-05-22

**Authors:** Matias Micheletto, Vinicius Petrucci, Rodrigo Santos, Javier Orozco, Daniel Mosse, Sergio F. Ochoa, Roc Meseguer

**Affiliations:** 1 Department of Electrical and Computers, Universidad Nacional del Sur, Consejo Nacional de Investigaciones Científicas y Técnicas (CONICET), Bahía Blanca 8000, Argentina; matias.micheletto@uns.edu.ar (M.M.); jadorozco@gmail.com (J.O.); 2 Department of Computer Science, Universidade Federal do Bahía, Bahía 40110909, Brazil; vpetrucci@ufba.br; 3 Department of Computer Science, University of Pittsburgh, Pittsburgh, PA 15261, USA; mosse@cs.pitt.edu; 4 Department of Computer Science, Universidad de Chile, Santiago 8370456, Chile; sochoa@dcc.uchile.cl; 5 Department of Computer Architecture, Universitat Politècnica de Catalunya, 08034 Barcelona, Spain; meseguer@ac.upc.edu

**Keywords:** Flying Real-Time Network, flying witness units, UAVs, real-time schedulability, communication support, disaster management

## Abstract

While there have been important advances within wireless communication technology, the provision of communication support during disaster relief activities remains an open issue. The literature in disaster research reports several major restrictions to conducting first response activities in urban areas, given the limitations of telephone networks and radio systems to provide digital communication in the field. In search-and-rescue operations, the communication requirements are increased, since the first responders need to rely on real-time and reliable communication to perform their activities and coordinate their efforts with other teams. Therefore, these limitations open the door to improvisation during disaster relief efforts. In this paper, we argue that flying ad-hoc networks can provide the communication support needed in these scenarios, and propose a new solution towards that goal. The proposal involves the use of flying witness units, implemented using drones, that act as communication gateways between first responders working at different locations of the affected area. The proposal is named the Flying Real-Time Network, and its feasibility to provide communication in a disaster scenario is shown by presenting both a real-time schedulability analysis of message delivery, as well as simulations of the communication support in a physical scenario inspired by a real incident. The obtained results were highly positive and consistent, therefore this proposal represents a step forward towards the solution of this open issue.

## 1. Introduction

Every year, natural and human-made disasters hit urban areas, producing dramatic losses in terms of human lives, damage to property, and psychological after-effects on the civil population. Examples of these extreme events are earthquakes, flash-floods, tsunamis, and landslides. Once the incident occurs, the relief efforts are typically focused on evacuating the civil population and conducting the search-and-rescue (SAR) process. This period lasts up to 72 h [1], and it is known as the golden relief time. During such a period, the SAR teams (which typically include firefighters and military personnel) scout the affected area, searching and evacuating as many people as possible. Given the short time period available to prepare and conduct these activities, the teams are deployed in the area as quickly as possible and without much planning, and then they are relocated according to the process needs. The incident commander (IC), who is in charge of the operations in the field, periodically evaluates the results of the relief activities, adjusts the SAR process plan, and assigns new goals to the teams. These activities require digital communication support between the teams, and also with the IC, since multimedia information should be shared among them (e.g., maps, pictures, and text/audio messages) in order to support decision-making processes at individual, team, and inter-team levels. Moreover, this communication support should be reliable and consider time constraints for message delivery (i.e., to have real-time characteristics) to allow the maintenance of activity coordination and thus improving the effectiveness of the response process.

The literature in this area reports a long list of limitations for using mobile phones and Ultra High Frequency/Very High Frequency (UHF/VHF) radio systems to support these processes [2,3,4], and it also recognizes the need to advance the research towards providing real-time and stable digital communication in the field, as a way to reduce the impact of these events on the civil population [5,6,7,8]. Examples of recent incidents where the response process was seriously limited by the communication support are the flood and landslides affecting Rio de Janeiro (Brazil) in 2011, where almost nine hundred people died [9], and also the wild fire of Yarnell Hill (USA) in 2013, where 19 firemen died trapped by the fire [10].

Several recent proposals report solutions that try to deal with this communication challenge, and most of them are based on opportunistic networks [6,7,8]. Although useful, they are limited to providing time-constrained message delivery, particularly when there is no direct communication between the teams that need to exchange information. This situation is almost the norm in disaster relief efforts.

Trying to address such a communication problem, and given that the teams are reallocated frequently, in a previous work the authors proposed the utilization of special devices named *witness units* (WUs) as intermediary network nodes [1,11]. The devices act as information repositories, allowing first response teams and also civilians to retrieve and deliver information depending on their roles. For instance, civilians could access the maps of the area and evacuation routes, while first responders could also retrieve or update information about the status of the SAR process in the area and the location of the SAR teams. These WUs could be attached to street light poles (or other static resilient locations) and then connected to the disaster management center through a satellite network. WUs rely on standard Wi-Fi network interfaces and provide access points for the human users. Although useful, the use of these units only partially addresses the challenge of providing time-constrained message delivery in the field, since WUs were initially conceived as stationary devices. Therefore, there is no way to ensure that two teams that require information exchange would be able to do so when required, either through a direct interaction between them or by using a WU as intermediary. Therefore, the authors also introduced the concept of *mules* [1,12], as mobile WUs that provide real-time guarantees of message delivery between the SAR teams and the IC. These mules can be implemented by attaching computing devices to vehicles (e.g., cars, motorcycles, bicycles), and they complement the service provided by regular WUs. For instance, the route of the mules can be designed to allow information exchange not only among teams, but also among WUs.

Although the use of land mules seems to solve the problem when the deadlines for the message delivery are not short, the orography of the terrain and the layout of the area usually reduce the chances to accomplish with short deadlines when required (e.g., when evacuation alarms are delivered).

In this paper, we address this challenge by introducing the idea of deploying a network of flying witness units (FWUs) implemented using unmanned aerial vehicles (UAVs, also called drones). These autonomous vehicles are usually designed to primarily collect data to meet a multitude of different tasks and applications. The lack of an onboard pilot allows them to exist in a variety of sizes and forms, which offers a dizzying array of options for organizations involved with disaster relief and mitigation.

These flying units can be specialized to play both of the previously discussed roles,  acting as regular witness units that provide a communication link among members of a SAR team, and also as flying mules that follow a path to provide communication among disconnected teams. Moreover, the use of these units allows the communication and mobility constraints imposed by the orography of the terrain and the street layout to be reduced, and the whole network can be seen as a FANET (Flying Ad-Hoc Network) [13]. While there have been several recent research efforts on FANETs, the design and efficient deployment of these networks still represents a great challenge. In disaster scenarios, the deployment of the FANETs needs to address the real-time behavior and also show the feasibility of timely message delivery. In this sense, the proposal reported in this article also makes a contribution to this study area.

This paper presents three main contributions. The first is the extension of the concept of witness units from stationary devices and land mules to flying witness units that have capacity to store-and-forward digital information between stationary and mobile teams (e.g., between SAR teams and the IC). The second contribution is the general structure proposed to implement a Flying Real-Time Network (FRTN) and the indications for its deployment in order to have a predictable behavior in message transmissions. The third contribution is the computation of real-time feasibility conditions for this kind of network, where there is no continuously available end-to-end path. This last contribution allows reducing the effort of prototyping these networks and evaluating their performance, since an analytical approach can be used instead of the typical network simulations. This article is an extended version of the proposal presented in [12].

The next section presents and discusses the related work. Section 3 introduces the system proposal, its structure, and the message transmission model. Section 4 proposes two meta-heuristics to minimize the deployment of nodes in a FRTN, while keeping the required connectivity. Section 5 presents the real-time analysis of the network model, showing that the FRTNs are able to provide stable and real-time digital communication in disaster relief scenarios. The analytic results are then compared to those obtained from simulations, thus corroborating the communication capabilities of these networks. Section 6 discusses the proposal considering real constraints, and finally Section 7 presents the conclusions and future work.

## 2. Related Work

In order to properly analyze the suitability of communication technology that supports disaster relief efforts, it is important to remark that these mitigation processes involve several phases, and the technology that is suitable to support one of them is not necessarily suitable for the others. These phases are *preparedness*, *response*, *recovery*, and *learning* [14]. The problem addressed in this paper is limited to the communication support required during the response phase, particularly in the “golden relief time” (i.e., the first response), where there is almost no time to transport or setup large communication infrastructures, and where regular communication services (e.g., wired and cell phones) and power networks are usually unavailable. In this sense, the infrastructure of the communication media should be easy to deploy, allow the mobility of the users, and support intra- and inter-organization interactions. Considering these restrictions, it is evident why land mobile radio (LMR) systems are the most frequently used communication support in this period of the response process [15,16].

The literature also reports several limitations of analog communication media to support this process (e.g., their inability to transmit digital information, control the access to shared information, and ensure the availability of a communication channel when required) [2,3,4]. For that reason, previous research works have proposed several kinds of mobile ad-hoc and opportunistic networks to try provide real-time and stable digital communication during disaster relief efforts. These proposals typically complement the use of LMR systems. For instance, a human-centric wireless sensor network was introduced in [1] to improve communication and the availability of critical information during SAR activities. While the added layer of human sensors can help input extra information into the network, the underlying deployed network allows such information to be shared, and therefore becomes an essential component for the success of the communication process. Then, in [17], the authors extend the previous work and propose a human-centric approach to model IoT-based solutions for man-made incidents such as train derailments or terrorist attacks. Both proposals consider the simultaneous use of stationary witness units (WUs) as well as land mules. These mules can be implemented using manned and autonomous vehicles, and their main role is to ease the exchange of information among working units (e.g., response teams, the IC, and WUs) when there is no direct communication path between them.

Trying to deal with the dynamism of these networks and increase the usefulness of WUs, the NASA Glenn Research Center developed the Unmanned Aircraft System (UAS) to select and test a communication technology for the UAS Control and Non-Payload Communications (CNPC) link [18]. This organization evaluated the performance of several potential technologies for the CNPC link through the use of detailed software simulations, and LTE and 802.16 ranked as the top two. Similarly, the SMAVNET (Swarming Micro Air Vehicle Network) project at the École Polytechnique Fédérale de Lausanne (EPFL) introduces a flying ad-hoc self-organizing network that uses swarming communication protocols to build an alternative wireless network for rescuers in emergency scenarios [19]. This initiative proposes the use of a kind of self-organizing mesh network, where the UAVs rely on multi-hop communications to better cover the operation area. Although interesting, the full deployment of FANETs at large scale may be too intricate in disaster scenarios, given that it would require dealing with non-trivial decisions such as dynamic routing [20].

In [21] the authors claim that there have been no practical solutions, outside of military applications, to quickly provide digital communication support in disaster relief scenarios. Consequently, they propose an autonomous system to deploy UAVs as the first phase of the disaster recovery communication network for wide areas. An automation algorithm controls the deployment and positioning of a set of quadcopters (UAVs), based on a hexagonal pattern distribution that uses an open source MAVLink point-to-point communication protocol. The distributed execution of the algorithm is based on a centralized management of UAV cells, which involves assigning the role of supernodes to some specific UAVs. The use of open-source solutions makes the system easily reproducible and accessible, but at the same time it exposes the system to several types of network attack. Therefore, this solution requires extensive protocol modifications and additions to become robust and reliable [22].

As an alternative to the previous works, this paper proposes a Flying Real-Time Network (FRTN) to provide support to SAR teams and the IC during first responses to natural and man-made disasters. We introduce a feasibility analysis of the proposed network model based on the demand bound function and divide the message propagation in different stages, each one with a computed release time and deadline. To the best of the authors’ knowledge, this kind of analysis has not been done before for ad-hoc networks, and particularly for FANETs. Therefore, this proposal represents an advancement of the state-of-the-art in this study domain.

## 3. Proposed Network System

We argue that the use of UAVs has several advantages for the implementation of an alternative network system that provides communication in a disaster environment. For instance, it can help reduce the risks assumed by rescue teams and enhance the teams’ effectiveness, while providing viewing angles that are impossible to obtain with other types of mules. The cost of these vehicles is falling, and their technological capabilities are rapidly increasing, including services such as automated obstacle avoidance, route planning, point-to-point navigation using way-points, and tracking points of interest. All these features facilitate the autonomous deployment of the network and thus the rapid provision of the connectivity required by the participants of the first response process.

UAVs may become the primary form of information capture and transportation in disaster scenarios. Mostly used for video surveillance, UAVs equipped with data communication and software capabilities can share data through a collaborative architecture, thus allowing an efficient FANET to be built. These vehicles hold a great potential when their collected data is integrated with geographic information systems and crisis management tools. Considering that drones may have different sizes and flight autonomy, a proper selection and distribution of these vehicles is necessary to provide the best network coverage in the disaster area, and also an efficient communication support to the search-and-rescue activities.

Given the constraints considered in this proposal for message delivery, we have called these particular FANETs “Flying Real-Time Networks” (FRTNs). Next, we describe the types of flying witness unit (FWU) that can be present in a FRTN, and the way in which these units should be deployed in the work area to try maximizing the communication support and minimizing the number of intermediary participants.

### 3.1. Flying Witness Units

**Figure 1:**
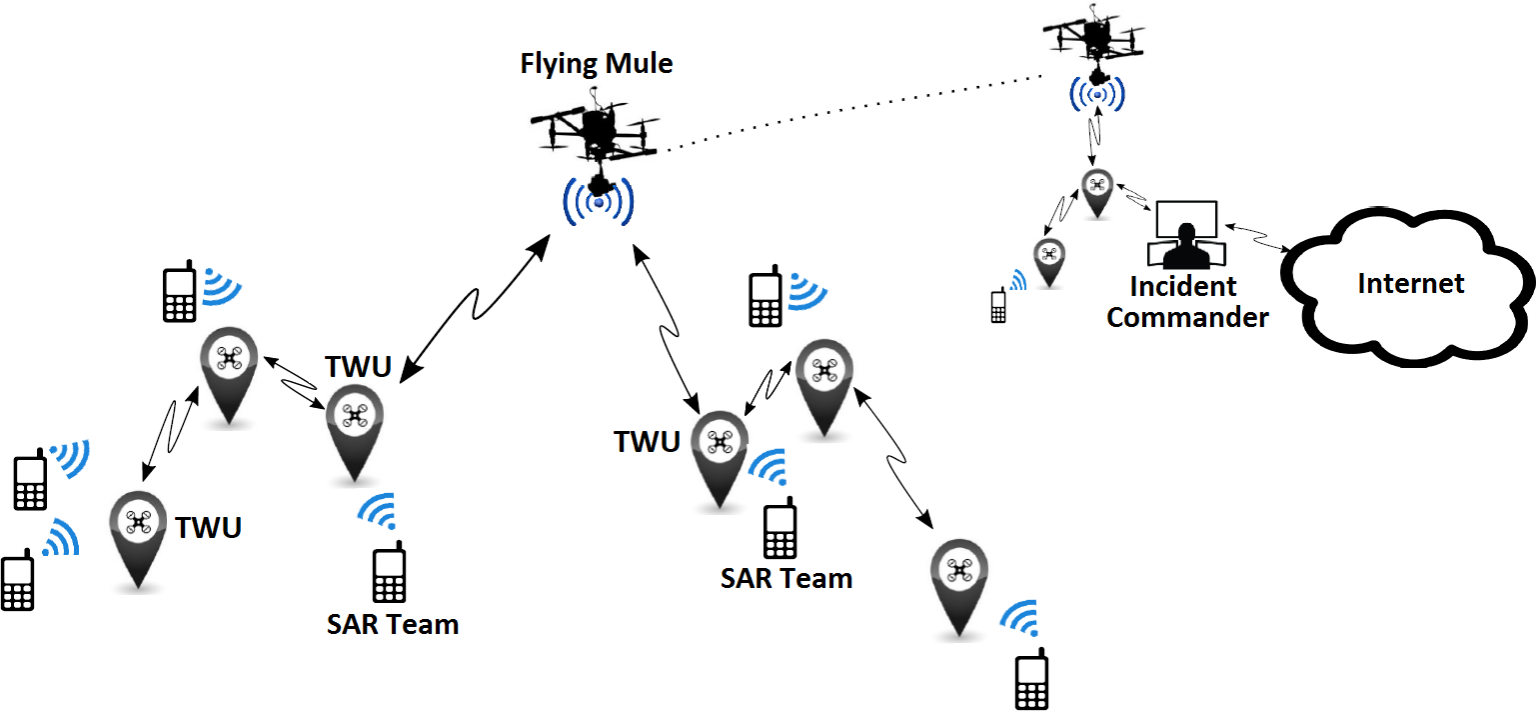
Example of an FRTN that includes eight team-centered witness units (TWUs) and two flying mules. Members of search-and-rescue (SAR) teams are depicted by smartphones, the “location” symbols near these devices are the TWUs, and the helicopters represents the flying mules.

A FWU is an unmanned mobile unit that locally stores information and transfers it to other network nodes (e.g., SAR team members) at the same or other locations. Figure 1 shows an example of an FRTN that supports a first response process, and illustrates the main structure and dynamics of these networks.

In order to support the message delivery in a FRTN, this proposal considers two kinds of FWUs. The first is mainly used to provide direct contact among the members of an SAR team. In this case, each team counts with a dedicated FWU that follows it and acts as gateway to interact with the FWUs of other teams. These units, named team-centered witness units (TWUs), have a mobility level similar to the teams they follow; typically, this means low mobility. If we increase the abstraction level in the analysis and consider a TWU as the communication device of an SAR team, we can conceive meta-TWUs potentially able to bridge regular TWUs. These meta-TWUs are governed by the same rules and have the same purpose and behavior as the regular TWUs, and therefore this proposal does not differentiate between these two type of TWU.

The second type of FWU acts as a flying *mule* that goes from one location to another, following a path that allows it to link disconnected teams. Therefore, the challenge of providing a communication link between disconnected TWUs can be addressed by using meta-TWUs, mules, or a mixture of the two. The best solution will depend on the distance between the nodes to communicate and the communication range of the technology used to support these information exchanges. The goal is always to minimize the number of witness units (i.e., TWUs or mules) required to cover the work area with a signal quality that allows first responders to count on real-time message delivery. Thus, the FRTN saves energy and increases the time period of communication support.

In the example shown in Figure 1, the cell phones represent the devices used by members of SAR teams to support their activities. These devices use Wi-Fi links (or any other technology) to communicate with the TWUs, which in turn communicate with the flying mules participating in the FRTN. The mules are represented as helicopters in Figure 1. Thus, the FRTN allows the information exchange among teams and with the IC. An added value of the FRTN is that civilians could also access the network, for instance to ask for help or receive directives from SAR teams near them. This contributes to make the SAR process more expedited and effective.

We envision the TWUs landing at certain spots, and then flying again following the movements of their corresponding SAR teams. The stops of these units allow extending the time window available for exchanging messages and also saving energy. We also envision that only the FWUs triggering the response process will be remotely controlled, particularly those carrying TWUs to be deployed to support SAR teams. Thereafter, truly autonomous behavior of TWUs and flying mules is the goal.

### 3.2. Initial Network Deployment

Network deployment is a key issue in emergency situations, since the communication network cannot be set-up ahead of time for two main reasons. First, most natural disasters typically destroy or damage the physical infrastructure in the affected area, thus jeopardizing the availability of these networks when required. Second, it is not clear where one should deploy the new communication network ahead of time, given that in most cases it is impossible to predict what areas will be affected by the event.

Once the extreme event has taken place, the IC decides where the SAR teams should be deployed first in order to start the relief activities; in that case, a TWU is assigned to each SAR team. These units will probably not be sufficient to cover the transmission range between the teams and the IC, and therefore additional FWUs (particularly meta-TWUs or flying mules) should be used to link the SAR teams with the IC.

**Figure 2:**
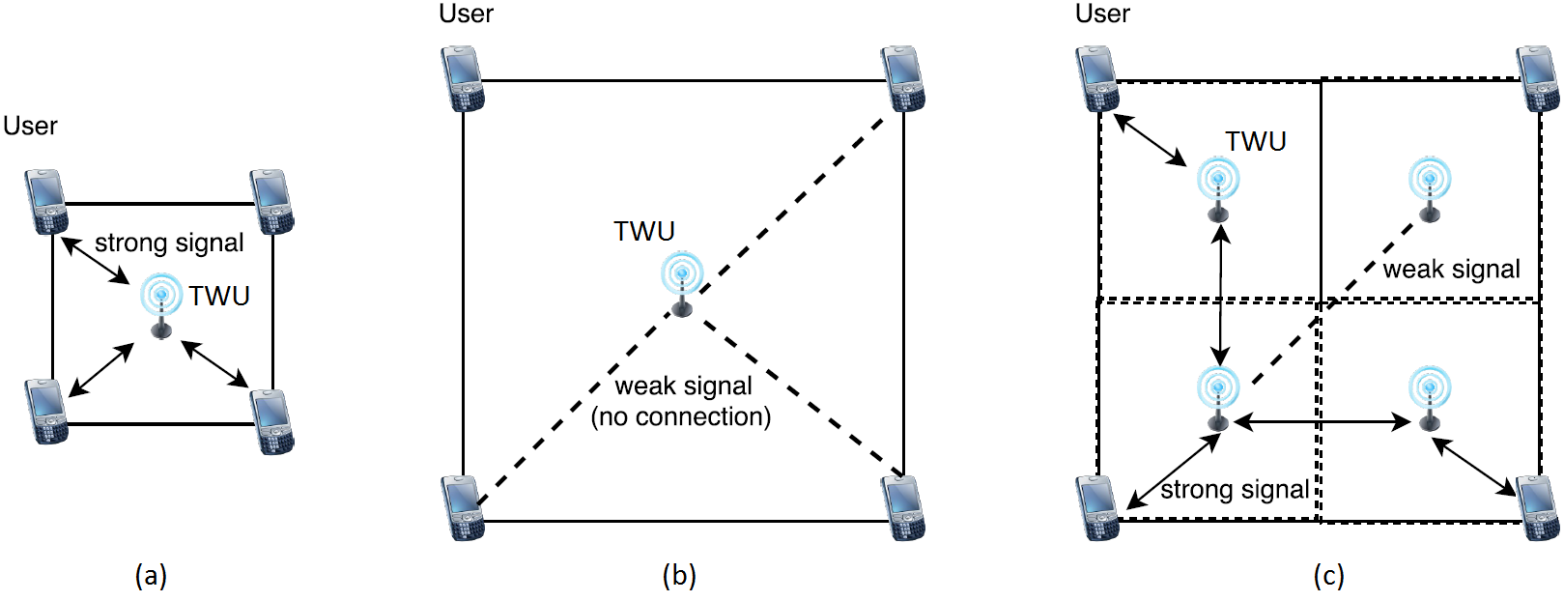
TWUs deployment scenarios: (**a**) small area with a single TWU providing communication to members of a SAR team; (**b**) large area supported by a single TWU; in this case, nodes count on weak or no communication signal to interact among them; and (**c**) large area with four TWUs that can provide adequate communication with bounded delay; this is an example of a feasible solution to cover large areas.

Next we describe a solution to the problem of determining a suitable deployment for the TWUs, given a certain physical area and communication range. As expected, we would like to deploy the TWUs in a specific set of locations, so that a minimal number of TWUs are used to guarantee communication in a given geographic area. We can assume as input a squared work area specified by adjustable side parameters. This matches with the typical quadrants used in first responses to divide the scouting area during SAR activities. Figure 2 shows various TWU deployment scenarios and also their network coverage areas.

The challenge of performing an appropriate deployment of TWUs can be reduced to a clustering or continuous/unconstrained location problem, where the facilities (in our case, the TWUs) can be anywhere in the plane [23], which is NP hard. This means that we can only have an approximation to the solution in polynomial time; conservatively, we can deploy extra FWUs to meet communication requirements. Thus, we can increase the network robustness (i.e., its tolerance to failures), a feature appreciated in highly dynamic and uncertain environments, such as in FANETs.

We solve the initial deployment using an adaptation to the widely used *k*-means algorithm (Lloyd’s algorithm) [24,25], which has average running time complexity of O(k×n×i), were *k* is the number of clusters, *n* is the number of data points, and *i* is the number of iterations required for convergence. In practice, the *k*-means algorithm is very fast and the number of performed iterations is effectively much less than the number of data points in the space. We chose the *k*-means algorithm because of its simplicity and short running time.

As described in Algorithm 1, the deployment works by clustering data points in a two-dimensional Euclidean plane. Given *P* user locations, the goal is to choose minimal cluster centers (TWU nodes), given by *Q*, that minimize the average distances between each data point and its closest center, while allowing nodes to communicate effectively by guaranteeing that the distance between each point (e.g., a SAR team) and its cluster is within a suitable transmission range (strong signal strength).

**Algorithm 1** Initial placement of TWUs in a given the emergency area
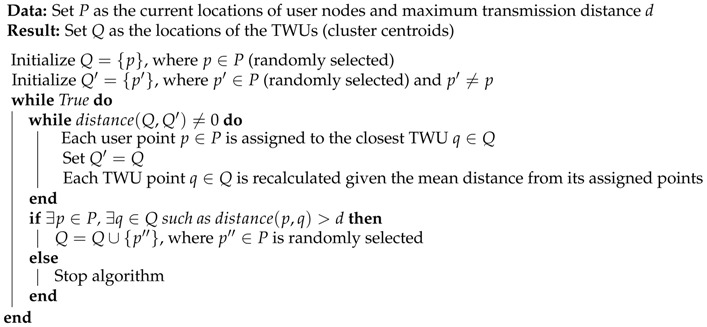


Because the proposed TWU deployment is a heuristic approach based on the *k*-means algorithm, it may fall in local minima. In order to address this situation, we used a common practice, which is to restart the deployment process many times with different initial conditions, up to a set limit, and then report the best result found. In our case, after restarting ten times the process was adequate to get useful approximated results for each deployment scenario.

Using a discrete event network simulator (sim2net) (https://pypi.org/project/sim2net/3.1.2/), we modeled an ad-hoc network consisting of a set of communicating nodes (in our case, SAR team members and TWU nodes), where each one moves according to a specific mobility pattern. Initially, no flying mules were considered to link disconnected teams, if any. The simulated area was a square of 1000 × 1000 m using a Wi-Fi antenna of 200 m range, where several numbers of TWUs were deployed. The algorithm for nodes placement uses as input the location of the teams to be supported, and the communication range of the technology utilized to do that; therefore, a change in the communication technology being used or in the location (or number) of teams to be supported represents only a change in the input parameters for the algorithm. It is important to remark that the goal of the algorithm is to identify the minimal number of TWUs, and their location, required to provide communication support to a set of teams in the field.  Moreover, the most suitable location for the TWUs should be adjusted periodically, given that the SAR teams change their location after finishing the operations in a certain quadrant.

The simulations considered that the communication among nodes was carried out by sending/receiving messages via the wireless links considered by the transmission model. A simplified path loss model was also used to allow these nodes to communicate directly when they were within a suitable transmission range of each other, considering a signal strength above the minimal operational level. This means that no packet is lost during communication and the message delay is bounded (the bound is known).

**Figure 3:**
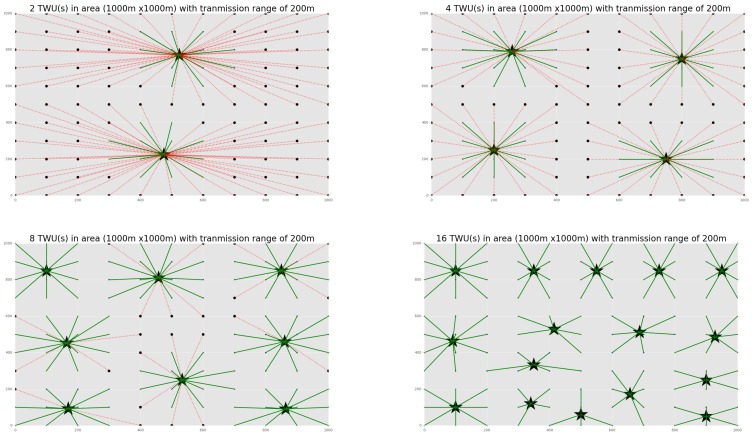
Simulation results considering various numbers of TWUs (2, 4, 8, 16) and particular allocations for them to provide stable and real-time digital communication support.

Figure 3 shows the several deployments that were simulated, and the number and location of the TWUs supporting the communication in the whole area. Each dot in the grid represents a potential end-user (e.g., a first responder) and the color of the lines indicates the quality of the communication link between the TWU and each end-user; green lines indicate a communication quality good enough to support message exchange, and red lines means poor-quality links (or no communication).

The simulation results show that 16 TWUs were required to fully cover the work area without any loss in terms of connectivity; however, 8 units were enough to address at least the 80% of the communication requests. Considering these results and the fact that several communication instances can be happening simultaneously between a TWU and a set of first responders, we can evidently expect that the use of these networks actually provide the stable and real-time digital communication support required in the study scenario.

Algorithm 1, used in these simulations to perform the initial placement of TWUs, represents a first approximation to help generate variations in the TWUs’ locations for more complex deployment scenarios. In that direction, the next section presents two meta-heuristics to search for sub-optimal deployments of the network nodes, in order to reach maximum communication coverage in the current work area, using the minimum active number of nodes and energy consumption.

## 4. Network Deployment Model

During the deployment of the network, the initial locations of the nodes must be defined as described in Section 3.2. The network topology should contain the minimum number of TWUs, and the total length of the network links must be short enough to guarantee a low communication delay between the SAR teams and the IC. At run-time, the power consumption of all mobile units (which is directly related to the network deployment) becomes an important aspect to consider, since it allows  the communication service to be kept active for longer time periods—hopefully for the whole golden relief time, although this would typically involve the exchange of units.

In order to represent a realistic scenario for studying disaster relief activities, we consider a set of $N_{f}$ nodes, each one with a fixed location. One of these nodes plays the role of IC and the rest are SAR teams. The teams and the IC have a communication range defined by $Pf_{i}$, for $i=1,...,N_{f}$. Each team must communicate with the IC, but eventually the communication range between them is not short enough to perform a direct interaction, and therefore the signal must be repeated by TWUs (particularly, by meta-TWUs) or eventually the information should be transported to the IC and vice versa using mules.

Formally, let *X* be a matrix of $N_{m}$ rows by three columns containing the positions of $N_{m}$ mobile nodes (SAR teams). $X_{i}$ is the position of the *i*-th node of the set. The third component of *X* represents the altitude of the node location. Because the mobile nodes are on ground, the altitude of its location is assumed as a function of the first two components of *X*. Let *Z* be a matrix containing the elevation map of the terrain where the network must be deployed. Because *Z* is discrete and the mobile nodes can be located in any position within the area, bi-linear interpolation is used to calculate the altitude of each node position.

We consider that each node has a maximum communication range defined by $Pm_{i}$, with $i=1,\;...,\;N_{m}$. To establish a link between two nodes, they must be in range and in line of sight. For a node *i* to be linked to a different node *j*, this distance can be expressed as follows:

(1) $$\mathrm{dist}\left(X_{i},X_{j}\right)<\min\left(Pm_{i},Pm_{j}\right)\wedge \mathrm{los}\left(X_{i},X_{j},Z\right),$$

where dist(v,w) is the Euclidean distance between *v* and *w*, and los(v,w,z) computes the line-of-sight visibility of points *v* and *w* according to elevation map *z*. Taking these conditions into account, if random allocation of mobile nodes is used, $N_{m}$ must be a sufficiently large number such that the communication between the SAR teams and the IC can be achieved.

**Figure 4:**
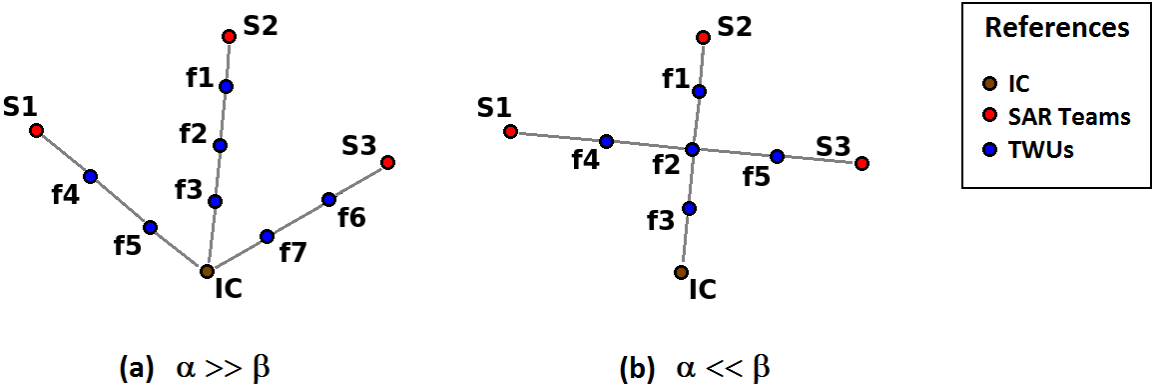
Network deployment situations: (**a**) more nodes using minimal paths and (**b**) less nodes with longer paths. IC: incident commander.

We further extend this network model to consider a communication protocol using a simple routing algorithm that determines the path of minimum length between each SAR team and the IC. Each path is enumerated as $path_{i}$ with $i=1,...,N_{f}-1$. Figure 4 illustrates two network deployment situations. For each team, there must be a route that allows communication with the IC. Discarding nodes that are not part of any of the $N_{f}-1$ routes, we obtain the minimum number of nodes for the *X* distribution, which is named $N_{opt}$. Therefore, it is necessary to determine *X* such that $N_{opt}$ is minimum, and the sum of the distances of each path that links the SAR teams with the IC is also minimal. Then, the optimization problem is formulated as follows:

(2) $$\mathrm{minimize}f\left(X\right)=\alpha \cdot \sum_{i=1}^{N_{f}-1}\mathrm{length}\left(path_{i}\right)+\beta \cdot N_{opt},$$

where α and β allow us to weigh the importance of minimizing the number of TWUs or maximizing the speed of the network by reducing the distance of each path. Although the terms of Equation (2) are independent, it is assumed that the smaller the number of nodes, the less flexible the network is to allow direct paths from the SAR teams to the IC, and therefore the length of each path is greater.

### 4.1. Adaptive Topology Based on Power Consumption

As mentioned previously, power consumption is a critical aspect in the operation of the TWUs, since it determines the time period in which these devices will be able to provide the communication service. We assume that the dynamics of the power required for each TWU is proportional to both the number of retransmitted messages and the distance to the linked neighbors. In order to balance the power consumption of the TWUs when using the network, it is important to make sure that the higher the communication load of a link, the lower the distance between the joined nodes.

Assuming that the increase of the load of different links is considerable and the information loading process lasts for relatively long periods, we propose a distributed model in which the network adjusts its topology according to these variations, and where each TWU must update its position following Equations (3) and (4):

(3) $${\vec{v}}_{i}=b\cdot {\vec{v}}_{i}-\sum_{j\in links}k_{j}\left({\vec{x}}_{i}-{\vec{x}}_{j}\right),$$

(4) $${\vec{x}}_{i}={\vec{x}}_{i}+{\vec{v}}_{i},$$

where ${\vec{x}}_{i}$ is the position of the *i*-th TWU, and ${\vec{v}}_{i}$ models the attractive forces that act on each TWU and point towards the neighboring nodes. The values of the constants $k_{j}$ must be calculated according to the data rate through the *j*-th link. The *b* constant allows adjustment of the simulation establishment time in order to stabilize the positions of the TWUs as quickly as possible.

**Figure 5:**
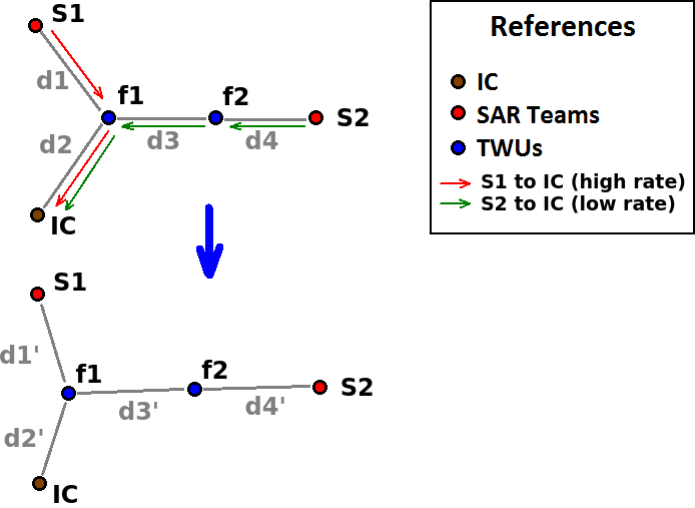
Dynamics of the adaptive network model.

Figure 5 shows an example of a network deployment, where an increased load in the sending of messages between team S1 and the IC makes it so that TWUs f1 and f2 have to move to the left and slightly down. This reduces the distances d1, and in greater quantity d2, in order to keep the communication capability among the nodes. Naturally, the distances d3’ and d4’ will be greater than the original distances d3 and d4.

### 4.2. Performance Evaluation

**Figure 6:**
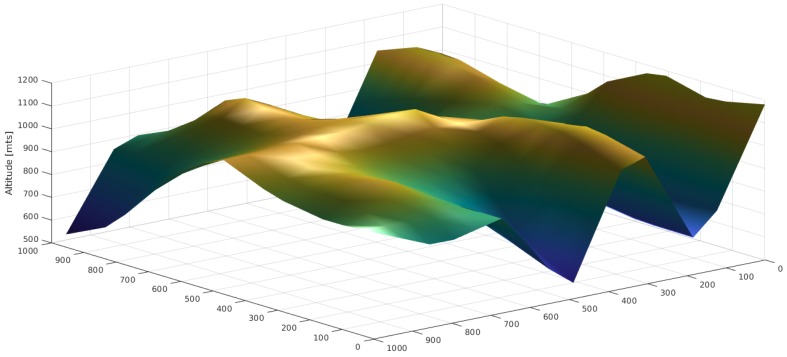
Terrain elevation map considered in the deployment example.

In order to evaluate the performance of the proposed algorithm for the deployment of TWUs, we assumed a terrain with elevations as shown in Figure 6. The orography of this physical scenario was inspired in the area affected by landslides in Rio de Janeiro (Brazil) in 2011 [9]. As part of the first response process, it is required to create a network of nodes that allows the SAR teams to be connected with the IC. These nodes were located in the positions shown in Table 1, referred to as the total deployment area.

The network nodes had a radial communication range equivalent to 20% of the width of the deployment area. The parameters chosen were α=1 and β=1; moreover, optimizations based on genetic algorithms (GAs) and particle swarm Optimization (PSO) were used to iteratively improve the nodes’ deployment in order to attempt to reach the optimum setting. These optimization methods were implemented in the programming language GNU Octave version 4.0, and the implementations were run in a personal computer with a Intel Core i7 7700hq @2.80 GHz processor, 16 GB RAM, and a Nvidia GeForce GTX1060 graphics processor.

Both PSO and GA require the generation of an initial population from which each algorithm evolves by improving the deployment solution, which in any case is sub-optimal. As mentioned before, random initialization can have a high computational cost due to the large number of nodes required to achieve connectivity between the SARs and the IC. To improve the initialization time and reduce the total execution time of the optimization methods, the deployment algorithm proposed in Section 3.2 was used as initial condition; small changes in these nodes positions were then introduced to represent variability in the initial setting.

When the GA-based optimization was used, the algorithm execution was limited to 3000 s, reaching a total of 173 generations, and the best deployment solution included nine TWUs. On the other hand, the PSO algorithm finished its execution after 2978 s, completing 200 iterations and the best solution considered 11 TWUs. The scores of these best solutions were 16.439 and 18.368 for GA and PSO respectively.

**Figure 7:**
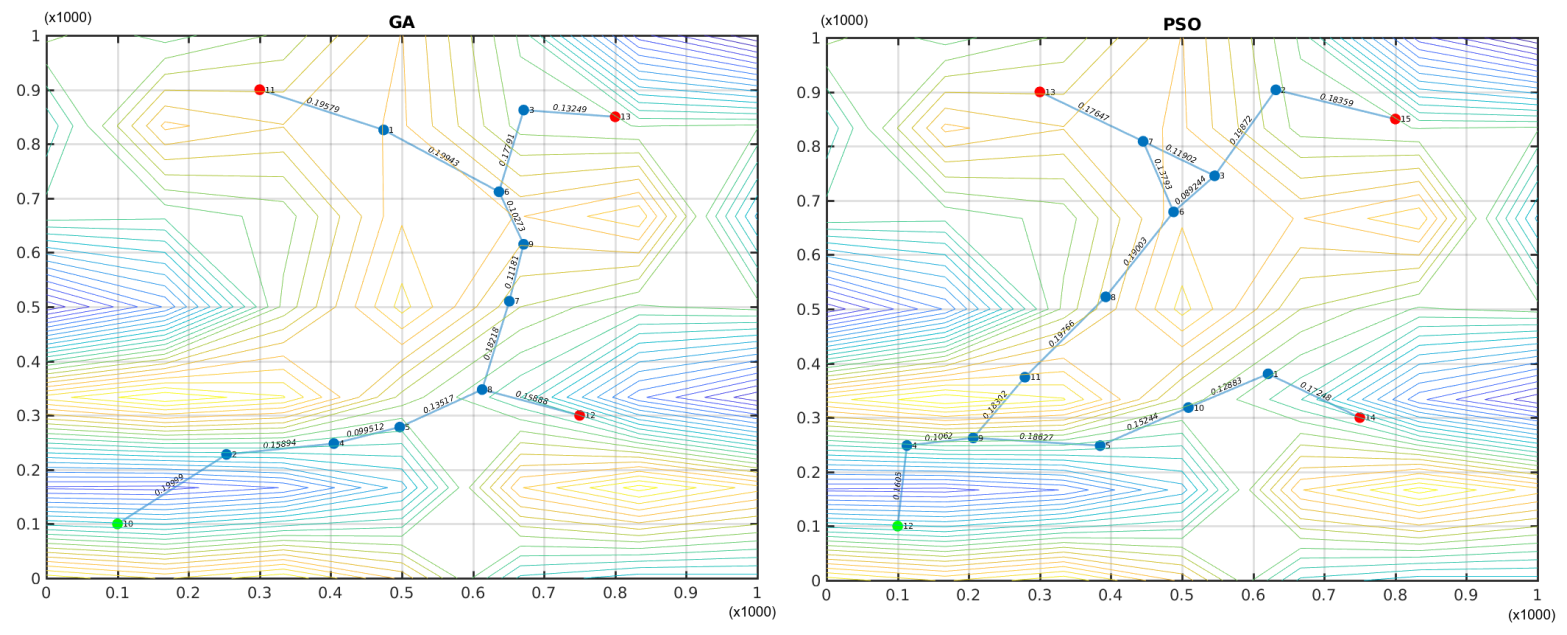
Sub-optimal network deployments generated by genetic algorithms (GAs) (**left**) and particle swarm optimization (PSO) (**right**). Green nodes represent the IC, red nodes are SAR teams, and blue nodes are TWUs.

The plots shown in Figure 7 indicate that the best deployment of TWUs for both GA and PSO was done surrounding the sloped area. This is because the nodes have to be close to each other in order to achieve visibility in mountain or valley zones; this significantly increases the network size.

**Figure 8:**
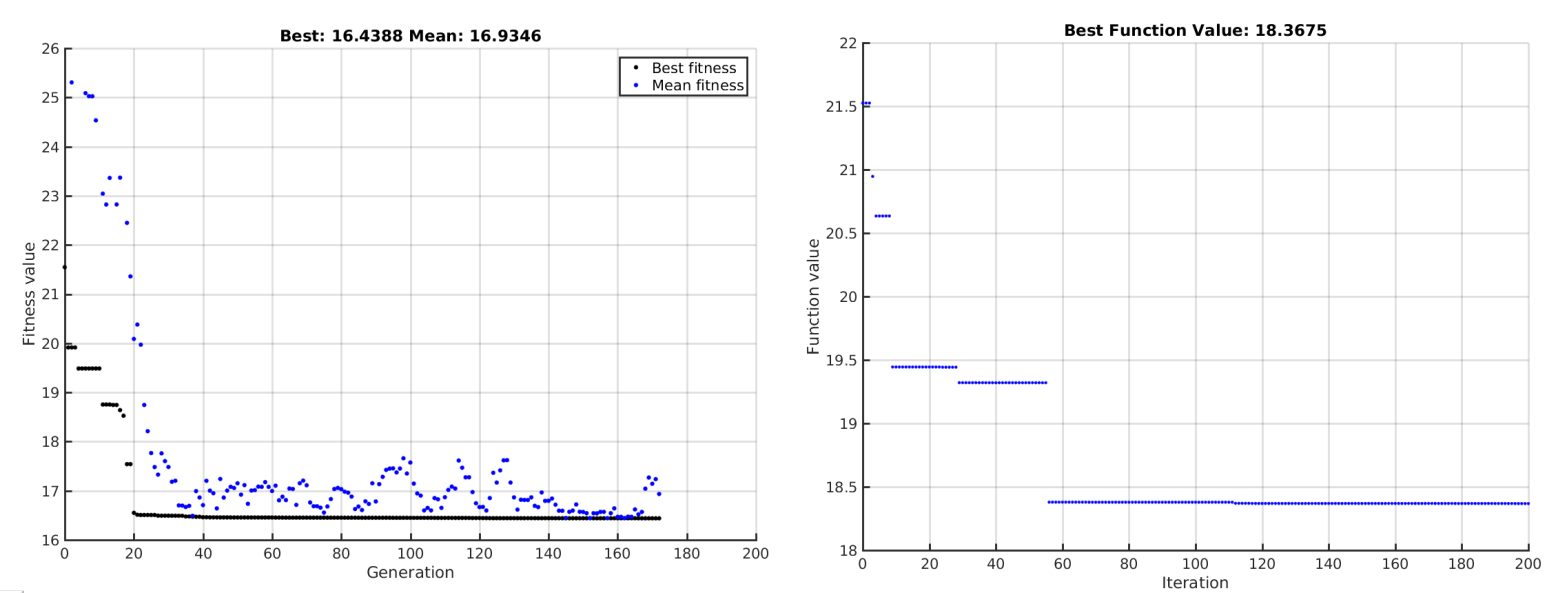
Historical evolution of the best deployment of TWUs when genetic algorithms (GAs) (**left**) and particle swarm optimization (PSO) (**right**) were used.

Figure 8 shows the historical best fitness value of each algorithm. The left plot displays the population best and mean fitness quality of each generation and the right figure shows the fitness function value of the best particle in the swarm for every iteration. It can be seen that PSO required fewer iterations to achieve the best solution compared to GA, but it tended to stagnate while GA continued improving the quality of the solution, although this improvement was very small.

## 5. Real-Time Analysis

As mentioned previously, the network system proposed in this article is based on an FRTN, which is built with TWUs (and eventually mules) that link the devices used by members of SAR teams and the IC. From an operating point of view, the FRTN is an opportunistic network in which there is no stable path between any pair of nodes in the system. Particularly, it is a special type of FANET [13], where real-time messages must be delivered before their deadlines in order to be useful. For this to occur, it is necessary to have bounded end-to-end transmission delays, and therefore the worst-case can be compared with the message deadline to verify the feasibility of the real-time operation of the network. The challenge in this case is that the network delay cannot be determined beforehand, as the topology is variable and there are also variable physical constraints. Determining these operation conditions involves performing a typical schedulability analysis of real-time systems [26]. In this sense, a contribution of this paper is to extend this analysis to consider opportunistic mixed networks composed of mobile nodes, semi-stationary, and stationary nodes (i.e., mules, TWUs, and the devices of the IC (located at the command post), respectively).

**Figure 9:**
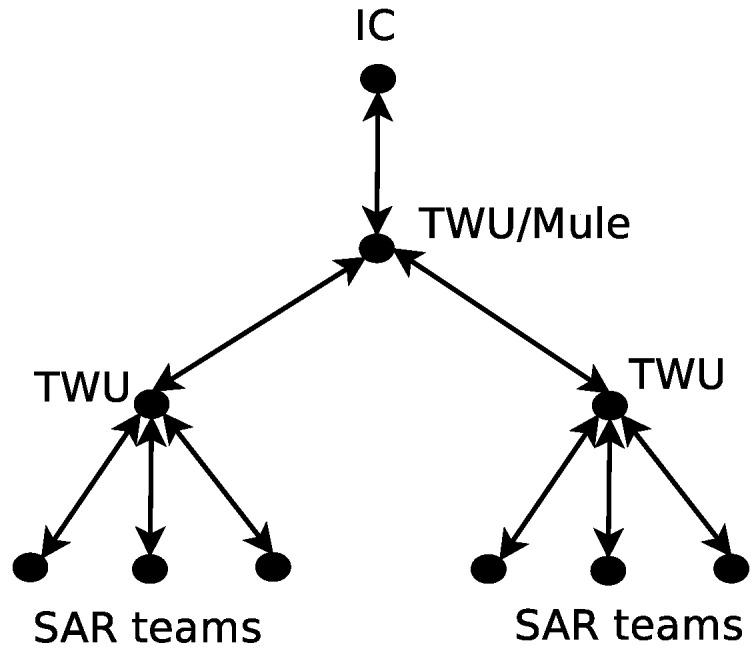
Hierarchical topology of a Flying Real-Time Network (FRTN) that supports first responses after a disaster.

When these networks support communication in particular application domains (e.g., emergency responses), they must consider the way in which such a scenario operates. For instance, the international search-and-rescue protocols establish that the communication between the SAR teams and the IC (or command post) should be performed hierarchically, as shown in Figure 9. Therefore, the IC typically delivers orders to SAR teams, and the latter return responses and awareness information to the IC. In this exchange, the sender transmits information to the closest TWU, which in turn acts as a gateway to mules or meta-TWUs that allow communication with the receiver.

### 5.1. Analysis of a Flying Real-Time Network

In FRTNs we consider a set of periodic and independent messages sent by the nodes (SAR teams). For simplicity of presentation, all messages have the same length, that is, they have equal payloads. Formally, a message $m_{i}$ originating in node *i* is described by the tuple 〈C,P,r,D〉, where *C* is the time required to transmit a message, *P* is the period, *r* is the release time, and *D* is the relative deadline of the message. The absolute deadline is computed from the release time and relative deadline, d=r+D. For simplicity, we consider that the time is divided in atomic units or slots. Events occur at the beginning of a slot. Time is denoted t=0,1,…,n.

**Figure 10:**
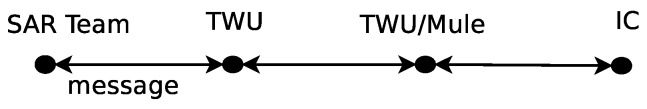
Message propagation pipeline.

In the network, the messages are generated in nodes and go up through levels before reaching the IC. In the reverse path, a message sent by the IC should traverse the same levels to reach an SAR team. Therefore, the transmission path can be seen as a “pipeline”, where each stage/level cannot process the message until it has been released by the previous one. Figure 10 shows the propagation model for a message considering that network topology.

Each stage in the transmission process requires a proper scheduling algorithm, and for each level, the real-time feasibility conditions should be verified for the system. Therefore, we must consider the moment in which the message arrives to intermediate nodes, and the latest instant at which it should leave that node. Let us suppose that there is one hop between the source and destination node. A message has a total delay of *x* slots if it is transmitted alone in the network, and as soon as it arrives to the intermediate hop, it is sent again. Let us suppose that the delay from the source to the intermediate node is *y* slots. If the message is delivered at r=0, it is not possible for that message to arrive before r=0+y to the intermediate node. In that case, the message should not leave the intermediate node after D−x−y in order to reach the destination node before its deadline. This means that the message should be processed in the intermediate node in at most D−x−y−r.

Considering the described scenario, we introduce the following notation to deal with the schedulability analysis in the intermediate nodes. For an intermediate node *j*, the release time of message *i* coming from node *i* is denoted $r_{i}^{j}$, the deadline is denoted $D_{i}^{j}$, and the transmission time is denoted $C_{i}^{j}$.

Although there are several algorithms for message scheduling, among the most used ones are rate monotonic (RM) and earliest deadline first (EDF). In the first algorithm, priorities are assigned to messages based on the message period, with shorter periods given higher priorities. At each opportunity, the message with higher priority is dispatched. In the second case, the highest priority is given to the message with the closest deadline. In that way, whenever the node has the chance to transmit, it dispatches the message with the closest deadline, therefore EDF can reach a better utilization of the network. Even though a different policy can be employed at each hop in the path, for simplicity, we assume an EDF scheduling policy for all of the stages.

It is known that EDF is optimal for scheduling a set of independent periodic messages within a network. However, in the problem proposed here, the message should traverse several hops before reaching its destination, and in each hop, a scheduling policy for message delivery is followed by the interim node. This affects the independent condition, as a message cannot be dispatched from node *j* if it has not left node *i* before. Thus, there is a dependency in the scheduling conditions of node *j* with respect to the scheduling conditions in node *i*. To overcome this problem, we use the Chetto algorithm [27] that transforms a set of precedence-related messages into a set of independent ones. In this way, a message with one hop is transformed into two independent messages being sent by two nodes, but with release times and deadlines modified for each of them. In this way, message $m_{i}$ going through node *j* to node *k* is seen as two independent messages, $m_{i}^{i}$ and $m_{i}^{j}$, that have equal transmission time, but different release times and deadlines. To accomplish this decoupling of the precedence relations, Chetto modifies the release times beginning from the first stage and the deadlines starting with the last one. Algorithms 2 and 3 present in pseudo-code the procedures for modifying the release times and deadlines, respectively.

**Algorithm 2** Chetto algorithm for modifying message release times*For modifying the release times:*
For any initial node of the precedence graph, set $r_{i}^{*}=r_{i}$.Select a message $m_{i}^{j}$ such that its release time has not been modified, but the release times of all immediate predecessors $m_{i}^{h}$ have been modified. If no such message exists, exit.Set $r_{i}^{j}=\max\left[r_{i},\max\left(r_{i}^{h}+C:m_{i}^{h}≺m_{i}^{j}\right)\right]$.Return to step 2.

**Algorithm 3** Chetto algorithm for modifying message deadlines*For modifying the deadlines:*
For any terminal node of the precedence graph, set $d_{i}^{*}=d_{i}$Select a message $m_{i}$ such that its deadline has not been modified, but the deadlines of all immediate successors $m_{k}$ have been modified. If no such message exists, exit.Set $d_{i}^{j}=\min\left[d_{i},\min\left(d_{i}^{k}-C:m_{j}≺m_{k}\right)\right]$.Return to step 2

As mentioned previously, in this proposal some drones are used as bridges, and therefore are in a fixed location, while others actually transport the messages between two nodes. This strategy introduces a delay that is not related with the transmission rate or computation power of the processor, but with the time needed by the UAV to transport the data between nodes *i* and *j*, $\Delta t_{ij}$. This delay can be considered constant and equal for every message between those two nodes. However, this delay affects the end-to-end transmission time and should be taken into account in the network analysis. The transportation delay must be subtracted from the deadline computed for that stage, as shown in Equation (5):

(5) $$d_{i}^{j}=d_{i}^{j}-\Delta t_{ij}.$$

Once the message transmission has been broken into different stages, the real-time feasibility test can be performed in each node of the “pipeline”. In order to do that, we use the demand bound function, $dbf(t_{1},t_{2})$ introduced in [28]. This test computes the worst-case work demand, and then it compares that metric with the time available in the system for message transmissions. For each stage or intermediate node $S_{i}$, if the available time is greater than the messages demand, the system is feasible in terms of real-time message delivery. Next, we formalize the definition of the dbf function for the interval $\left[t_{0},t_{1}\right]$, based on [28]:

**Definition** **1.** *The demand function for stage $S_{i}$, denoted by $dbf_{i}\left(t_{0},t_{1}\right)$, is the total time taken by all instances of the messages going through $S_{i}$, having release time and deadline within $\left[t_{0},t_{1}\right]$. For periodic messages, the demand function can be computed as follows:*

(6) $$dbf_{i}\left(t_{0},t_{1}\right)\overset{def}{=}\max\left(0,\sum_{m_{h}^{i}\in S_{i}}\left(\left\lfloor \frac{t_{1}-d_{h}^{i}}{P_{h}}\right\rfloor -\left\lceil \frac{t_{0}-r_{h}^{i}}{P_{h}}\right\rceil +1\right)C\right),$$

*where $d_{h}^{i}$ represents the modified deadline in stage j using the Chetto algorithm and Equation (5).*

According to [28], for any interval of length *t*, the $dbf(t_{0},t_{0}+t)$ is defined as the maximum possible demand in any interval of length *t*. Therefore, the dbf(t) is defined as follows:

(7) $$dbf\left(t\right)\overset{def}{=}\max_{t_{0}}dbf\left(t_{0},t_{0}+t\right).$$

Finally, we introduce the necessary and sufficient feasibility condition for the system as follows:

(8) $$\forall S_{i},\forall tdbf_{S_{i}}\left(t\right)\leq t.$$

Based on definitions (6) and (7) and the feasibility condition (8), the schedulability of the network operating as a real-time system can be determined. In order to do that, the scheduling condition should be verified for each stage in the network.

### 5.2. Computing the Scheduling Condition

In this section we explain how to systematically implement the feasibility test for the proposed FRTN. In this scenario we must consider that the use of mules introduces a transportation delay. Moreover, a mule has to fly from one spot (e.g., the location of a SAR team) to the next one to deliver the received messages and also collect new messages from SAR teams. The time required for doing this operation is usually much higher than the time needed to transmit information using a Wi-Fi protocol.

For the case of FRTNs, we have to extend the Chetto algorithm to include the maximum delay or worst-case transmission time associated to the stage. We are not analyzing the MAC protocol or routing at this time; rather, we focus on the maximum delay the message may suffer, and thus determine the feasibility of providing real-time message delivery in these networks. As previously mentioned, the message deadlines are modified backwards (beginning with the last stage), and the message release times are modified forwards (starting with the node originating the message).

Next we present an example that illustrates how the real-time condition is computed. Let us suppose an FRTN deployed after a landslide in order to support communication among three SAR teams that conduct search-and-rescue activities coordinated by an IC. Each team has 10 members and one TWU that acts as gateway to the mule. The network is composed of a set of mules for providing connectivity among the teams and the IC. Each node of an SAR team generates a message report and sends it to the IC every five minutes. These messages are forwarded by the gateway to the mule as soon as possible. Let us assume that each message contains 250 KB (or 2 Mb) of data (e.g., voice messages or pictures), and the bandwidth of transmission link with the TWU is 1 Mbit/s. We assume the messages have a simultaneous release at instant zero. Considering this condition, each message requires 2 s to be transferred from the node to the TWU. With 10 nodes operating with a round robin scheme, the TWU receives all the messages within 20 s; in this case, $r_{i0}=0$ for all $m_{i}$. Thus, each message has two hops to reach the IC and an absolute deadline of 600 s.

Let us suppose the flying time of the mules in the FRTN is 360 s. In the worst case, the flying mules require 60 s to deliver to the IC, the 30 messages collected from the TWU linked to the SAR teams. With these constraints, the available time for transmission is just 180 s. Applying Algorithms 2 and 3 to each message, we have 180 s for the last relative deadline and 160 s for the first one.

**Figure 11:**
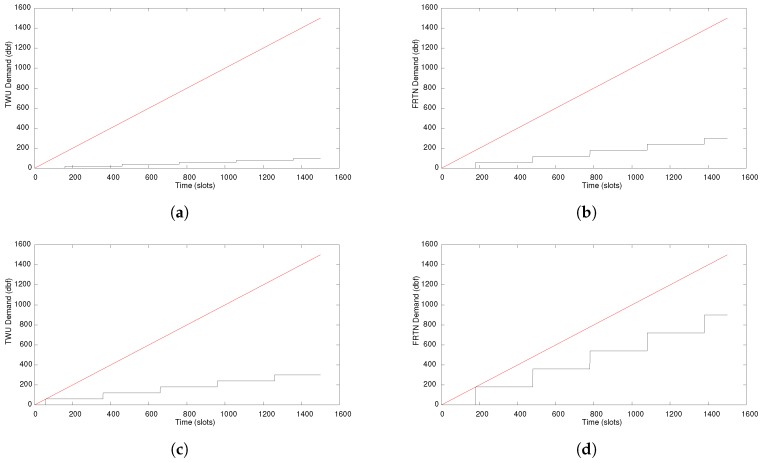
Demand bound function (*dbf*, in black) and *t* bound (in red) in the TWU and the mules. (**a**) TWU with 10 messages; (**b**) Mule involving 30 messages; (**c**) TWU with 30 messages; (**d**) Mule involving 90 messages.

Figure 11a,b present the dbf in the TWU and the mules, respectively. In both cases, the load is quite light and more traffic is possible. Figure 11c,d show the dbf in both hops when the number of messages being forwarded are tripled, and the results show that the system is still feasible and works within the proposed real-time parameters.

The previous example represents a simple case with just a few elements in the network. When the FRTN becomes larger and the messages have to do several hops before reaching the destination, additional problems appear (e.g., message routing). These cases should be considered at the moment of doing the real-time analysis of the network.

## 6. Discussion

The network system proposed in this work is flexible and general enough to be applied to support SAR activities in several natural disasters, such as landslides, floods, and earthquakes. The use of UAVs to support opportunistic communication opens the possibility of dynamically deploying a Flying Real-Time Network able to cover large scouting areas. The deployment of these FRTNs will depend on the characteristics of the disaster, the geographic features of the area, and the availability of UAVs to establish the most adequate FWU-based network design. The FWUs are specialized according to the role they play in the network (i.e., TWU/meta-TWU and mule). In order to address this deployment, we consider the following principles for the FWUs’ behavior:

- Each unit is independent and works on its own area.
- Each unit is independent, but it can work on overlapping areas (to decrease transmission time).
- Each unit can work on overlapping areas and it can also interact with an FWU in its neighborhood.
- Each unit works collaboratively and it is part of a large FRTN.

Several trade-offs should be considered depending on the operational areas of each FWU. The problem of determining the design of the best route to deploy and position the FWUs is an open problem. One critical issue for addressing this problem is considering the variability of the data transmission rate in wireless communication. This rate is much higher when the FWUs are closer to each other (up to 54 Mbit/s in 802.11 g), and much lower when the FWUs are far away (even lower than 1Mbit/s in 802.11g) [29]. for instance, an FWU with a buffer of tens of megabytes, flying at a speed of few tens of meters per second, could deliver messages to a network bandwidth over 1 Mbps, whereas a direct wireless link within a distance of few kilometers could deliver messages at a much slower rate (few tens of Kbps) [30]. However, direct links can deliver a single message with low latency, while an FWU may require a few minutes. The understanding of these trade-offs is key to taking advantage of the FWU network deployment [31].

We note that the FWUs should be deployed in such a way that the specific areas are covered with a satisfactory periodicity, and thus to effectively transfer the information from the SAR teams toward the IC. Real-time delivery of the messages can be best accomplished in cases where FWUs can work upon controlled mobility and predefined trajectories, or simply using as many meta-TWUs as required. The best alternative will depend on the availability of flying devices and the energy consumption of each solution.

## 7. Conclusions and Future Work

In this paper we argue for the use of UAVs to provide digital communication support in SAR activities during first responses to extreme events such as floods, landslides, and earthquakes. In these scenarios, and particularly during the golden relief time, ensuring the real-time delivery of digital information makes a difference, easing the coordination of activities and reducing the impact of these events on the civil population.

To the best of the authors’ knowledge, there is no proposal reported in the literature that provides stable and real-time digital communication support in the field during the golden relief time. In order to advance the state-of-the-art in this study domain, this article proposes the use of a particular type of flying ad-hoc network, named the Flying Real-Time Network (FRTN), that uses a set of flying witness units to link the stationary and mobile devices deployed in the field. This network supports the transmission of messages from the field to the incident commander, and vice versa, considering time-constraints.

The article also presents an analysis of these networks in order to prove real-time guarantees on the message transmissions. The results show that real-time requirements can be met by decomposing the message delivery in multiple independent stages. A synthetic disaster relief scenario, inspired by a real incident, was implemented using a discrete event network simulator. The simulation results corroborate the properties of the FRTN to support real-time message delivery. Therefore, this represents a step forward in the solution to a problem that has been difficult to address by the research community of the area.

The next step in this research initiative is to consider the message routing within the FRTN when a whole switching network is deployed to collect the shared information. This requires an important amount of TWUs to provide the necessary connectivity and reduce the amount of mules. In this scenario, the real-time analysis should include message congestion and energy consumption considerations.

## Figures and Tables

**Table 1:** Positions of the first response teams at the initial deployment stage.

Node	Coordinates (*X*,*Y*)
IC	(0.1, 0.1)
SAR 1	(0.3, 0.9)
SAR 2	(0.75, 0.3)
SAR 3	(0.8, 0.85)
